# Candidates for Synergies: Linear Discriminants versus Principal Components

**DOI:** 10.1155/2014/373957

**Published:** 2014-07-17

**Authors:** Ramana Vinjamuri, Vrajeshri Patel, Michael Powell, Zhi-Hong Mao, Nathan Crone

**Affiliations:** ^1^Department of Biomedical Engineering, Stevens Institute of Technology, Hoboken, NJ 07030, USA; ^2^Department of Biomedical Engineering, Johns Hopkins University, Baltimore, MD 21205, USA; ^3^Department of Electrical and Computer Engineering and the Department of Bioengineering, University of Pittsburgh, Pittsburgh, PA 15261, USA; ^4^Department of Neurology and Neurological Surgery, Johns Hopkins University, Baltimore, MD 21287, USA

## Abstract

Movement primitives or synergies have been extracted from human hand movements using several matrix factorization, dimensionality reduction, and classification methods. Principal component analysis (PCA) is widely used to obtain the first few significant eigenvectors of covariance that explain most of the variance of the data. Linear discriminant analysis (LDA) is also used as a supervised learning method to classify the hand postures corresponding to the objects grasped. Synergies obtained using PCA are principal component vectors aligned with dominant variances. On the other hand, synergies obtained using LDA are linear discriminant vectors that separate the groups of variances. In this paper, time varying kinematic synergies in the human hand grasping movements were extracted using these two diametrically opposite methods and were evaluated in reconstructing natural and American sign language (ASL) postural movements. We used an unsupervised LDA (ULDA) to extract linear discriminants. The results suggest that PCA outperformed LDA. The uniqueness, advantages, and disadvantages of each of these methods in representing high-dimensional hand movements in reduced dimensions were discussed.

## 1. Introduction

The central nervous system (CNS) is responsible for generating a potentially infinite set of hand postures used in everyday tasks such as grasping objects. It is hypothesized that the CNS reduces this computational burden by combining a discrete set of movement primitives or synergies [1, 2]. This allows high degree-of-freedom (DoF) control using a lower dimensional subspace [3]. Ninety percent of natural grasp movements produced by the human hand, which has greater than 25 DoF [4], have been reconstructed using five or six synergies [5, 6]. For these reasons, synergies have potential applications in dexterous control of prosthetic hands and have been recently applied to brain machine interfaces used in neural prosthesis [3, 7, 8]. However, deriving these synergies to effectively represent and reconstruct human hand movements poses a challenge. To address this challenge, several linear and nonlinear dimensionality reduction methods have been explored.

Nonlinear dimensionality reduction methods aim to reduce high-dimensional, nonlinear data to a lower dimensional manifold. Gaussian process latent variable model [9], geodesic trajectory generation models [10], Isomap [11], gradient descent method [12], and radial basis function [8] have been used to decompose upper limb movements either in kinematic or muscle space. Similarly, linear dimensionality reduction methods aim to transform a high-dimensional space to a linear low-dimensional subspace. Particularly successful in natural datasets, linear dimensionality reduction methods include nonnegative matrix factorization (NMF), principal component analysis (PCA), and linear discriminant analysis (LDA). Ajiboye and Weir [13] used NMF on mimed American sign language (ASL) postures to determine muscle synergies and found that subject-specific synergies were characterized by coactivation of muscles while population (similar across all subjects) synergies were typically dominated by a single muscle. PCA has been widely used to extract postural, kinematic, and muscle synergies [6, 14–17]. Mason et al. [14] used PCA, implemented with singular value decomposition, on a set of grasping tasks, and found that the first eigenposture resembled a whole hand grasp. This is similar to the results by [5, 14, 15]. Tresch et al. [18] compared multiple matrix factorization algorithms, including PCA, independent component analysis (ICA), NMF, factor analysis (FA), and combinations of the above, to determine muscles synergies. He found that the best performing methods (FA, ICA, NMF, ICAPCA, and probabilistic ICA) identified similar synergies. Dimensionality reduction methods not only yield significant eigenvectors from physiological data, but also reflect patterns in movement generation. Hence, two contrasting dimensionality reduction methods, such as PCA and LDA, may be partial towards different kinematic movement primitives. By comparing these two methods, we can determine which kinematic properties are most important in movement generation.

**Figure 1:**
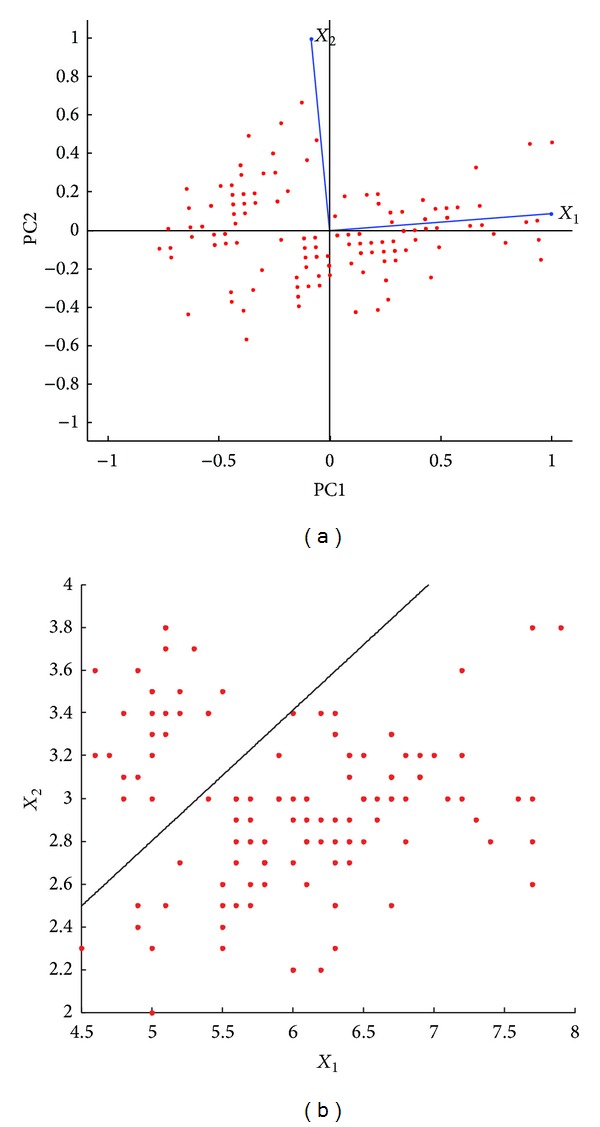
Comparison between PCA and ULDA on one sample data set. (a) Two-variable data was analyzed using PCA. The figure shows a biplot of principal components. The maximum variance is across the horizontal axis or PC1. The second highest variance is across the vertical axis or PC2. (b) The figure shows the linear discriminant that separates the two groups of data. The axes represent the two variables, *X*_1_ and *X*_2_.

PCA attempts to accurately represent the data in low-dimensional space by projecting the data in the directions of maximum variance as shown in Figure 1(a). PCA performed on a sample dataset is shown here. The first principal component axis corresponds to the direction of maximum variance of the data and similarly second and third in the decreasing order. LDA was used to classify the same sample dataset (Figure 1(b)). Directions of maximum variance as seen in PCA are not useful for classification. Hence, LDA projects the data in directions that preserve maximum separation or discrimination in groups or classes of data. LDA seeks dimensionality reduction while preserving as much class discrimination as possible.

In this paper, we compared two contrasting methods, PCA and LDA, in deriving synergies and evaluated them on a set of natural grasping and ASL postural movements. Both PCA and LDA are linear dimensionality reduction methods that reduce high-dimensional movements of the hand by looking for either most common patterns (PCA) or distinct separable patterns (LDA). While previous research has identified a limited number of discriminating muscles in a single synergy, such patterns have not been seen in kinematic space. As LDA aims to separate data, resulting synergies may reflect distinct patterns. Although LDA is a type of clustering method, it offers a direct contrast to PCA: separating variance (LDA) or maximizing variance (PCA). Thus, we can interpret the strengths and weaknesses of both methods. In order to maintain fairness in comparison with PCA, which is an unsupervised method, LDA used in this paper was also unsupervised (ULDA) first by labeling the data with k-means clustering and then classifying it using classical LDA.

## 2. Methods

### 2.1. Materials and Experiment

In the experiment, we used a CyberGlove (CyberGlove Systems LLC, San Jose, CA, USA) equipped with 22 sensors that captured hand movements at a sampling frequency of 86 Hz. In this paper, we only considered 10 of the sensors which measure the angles of the carpometacarpal (CMC), metacarpophalangeal (MCP) joints of the thumb and the MCP, and proximal interphalangeal (PIP) joints of the other four fingers. These 10 joints can capture most characteristics of the hand in grasping tasks. We used several objects of different shapes (spheres, circular discs, rectangles, pentagons, nuts, and bolts) and different dimensions (spheres: 1–5 cm in radius; discs: 2–10 cm in radius; rectangles and pentagons: 1–3 cm each side; nuts and bolts: 2–5 cm in length) in the grasping tasks. 10 subjects participated in this experiment after signing the consent forms approved by the Institutional Review Board (IRB) of the University of Pittsburgh.

A typical task consisted of grasping the above objects. Start and stop times of each task were signaled by computer-generated beeps. In each task, the subject was in a seated position, resting his/her right hand at a corner of a table and upon hearing the beep, and grasped the object placed on the table. At the time of the start beep, the hand was in rest posture, and then the subject grasped the object and held it until the stop beep.

In the first phase, subjects were instructed to rapidly grasp 50 objects, one at a time. This was repeated for the same 50 objects, and thus the whole training phase obtained 100 rapid grasps. Rapid grasping movements from the first phase were used in deriving synergies. We assume that by minimizing reaction time and minimizing continuous sensory feedback, the resulting task space is driven by synchronous weighted synergies resulting from impulses originating in a higher level of neural system. The rapid movement is achieved as a weighted sum of synchronous synergies. Only these 100 rapid grasps were used in extracting synergies using PCA and ULDA.

In the second phase, subjects were instructed to grasp the above 50 objects naturally (slower than the rapid grasps) and then this was repeated. So far, the tasks involved only grasping. In the third phase, to test the generalizability of the synergies over a broad range of postures, subjects were also asked to imitate 36 (10 numbers and 26 alphabet letters) American sign language (ASL) postures. The ASL postures were presented on sheets of paper. Here, subjects started from an initial posture and stopped at one ASL posture.

### 2.2. Preprocessing

First, we calculated angular velocities from the joint angle profiles collected in the experiment. We preserved only the relevant projectile movement—about 0.45 second or 39 samples under a sampling rate of 86 Hz.

Second, we constructed an angular velocity matrix *V* for each subject. Angular velocity profiles of the 10 joints corresponding to one object were cascaded, and each row of the angular velocity matrix represented one movement in time. The matrix had 100 rows (corresponding to 100 rapid grasping tasks from first phase) and 39 × 10 = 390 columns:

(1) V =[v11(1)⋯v11(39)⋯v101(1)⋯v101(39)⋮⋮⋮⋮⋮⋮⋮v1g(1)⋯v1g(39)⋯v10g(1)⋯v10g(39)⋮⋮⋮⋮⋮⋮⋮v1100(1)⋯v1100(39)⋯v10100(1)⋯v10100(39)],

where *v*_*i*_^*g*^(*t*) represents the angular velocity of joint *i* (*i* = 1,…, 10) at time *t* (*t* = 1,…, 39) in the *g*th grasping task (*g* = 1,…, 100).

### 2.3. Dimensionality Reduction

We then performed PCA and ULDA on the angular velocity matrix *V* composed of rapid grasping tasks to derive kinematic synergies.

*(1) Principal Component Analysis. *We implemented PCA using singular value decomposition (SVD). The angular velocity matrix *V* was factorized to three matrices *U*, Σ, and *S* as shown

(2) $$\begin{array}{c} V=U\Sigma S, \end{array}$$

where *U* is a 100-by-100 matrix, which has orthonormal columns so that *U*′*U* = *I*_100×100_ (100-by-100 identity matrix); *S* is a 100-by-390 matrix, which has orthonormal rows so that *SS*′ = *I*_100×100_; and Σ is a 100-by-100 diagonal matrix: diag⁡{*λ*_1_, *λ*_2_,…, *λ*_100_} with *λ*_1_ ≥ *λ*_2_ ≥ ⋯≥*λ*_100_ ≥ 0. Matrix *V* can be approximated by another matrix $\tilde{V}$ with reduced rank *m* by replacing Σ with Σ_*m*_, which contains only the *m* largest singular values, that is, *λ*_1_,…, *λ*_*m*_ (the other singular values are replaced by zeros). The approximation matrix $\tilde{V}$ can be written in a more compact form:

(3) $$\begin{array}{c} \tilde{V}=U_{m}\mathrm{diag}\left\{\lambda_{1},\ldots ,\lambda_{m}\right\}S_{m}, \end{array}$$

where *U*_*m*_ is a 100-by-*m* matrix containing the first *m* columns of *U* and *S*_*m*_ is a *m*-by-390 matrix containing the first *m* rows of *S*. Then, each row of *S*_*m*_ is called a* principal component* (PC), and the product *U*_*m*_diag⁡{*λ*_1_,…, *λ*_*m*_} is called the weight matrix.

For easy comparison, the elements of *S*_*m*_ in a way similar to (1) was written as

(4) $$\begin{array}{c} S_{m}\equiv \left[\begin{array}{ccccccc} s_{1}^{1}\left(1\right) & \cdots & s_{1}^{1}\left(39\right) & \cdots & s_{10}^{1}\left(1\right) & \cdots & s_{10}^{1}\left(39\right)\\ \vdots & \vdots & \vdots & \vdots & \vdots & \vdots & \vdots \\ s_{1}^{m}\left(1\right) & \cdots & s_{1}^{m}\left(39\right) & \cdots & s_{10}^{m}\left(1\right) & \cdots & s_{10}^{m}\left(39\right) \end{array}\right]. \end{array}$$

The angular velocity profiles (obtained by rearranging all joints row-wise for the PCs)

(5) $$\begin{array}{c} \left[\begin{array}{ccc} s_{1}^{j}\left(1\right) & \cdots & s_{1}^{j}\left(39\right)\\ s_{2}^{j}\left(1\right) & \cdots & s_{2}^{j}\left(39\right)\\ \vdots & \vdots & \vdots \\ s_{10}^{j}\left(1\right) & \cdots & s_{10}^{j}\left(39\right) \end{array}\right],\;j=1,\ldots ,m \end{array}$$

can be viewed as synergies. Six synergies accounted for 95% of variance in the postures.

*(2) Unsupervised Linear Discriminant Analysis. *Unsupervised linear discriminant analysis (ULDA) was implemented using a two-stage process. First, k-means clustering method created labels for the data. k-means clustering groups the data by calculating squared Euclidian distance between the data points. Each centroid is the mean of the points in that group or cluster. Classical LDA was then used based on these labels. ULDA was performed on the angular velocity matrix *V*. For *Y* = *W* ^*T*^ *V*, where *Y* is the transformed matrix and *W* is the transformation matrix, the optimal transformation using LDA is obtained by maximizing W* as follows:

(6) $$\begin{array}{c} W^{*}=\frac{W^{T}S_{B}W}{W^{T}S_{W}W}, \end{array}$$

where *S*_*B*_ denotes between class covariance and *S*_*W*_ denotes within class covariance. The linear discriminants derived from ULDA were used as new axes. The number of linear discriminants used was same as the number of principal components being used in comparison.

### 2.4. Reconstruction of Natural Grasping and ASL Postural Movements

The synergies extracted from PCA and ULDA were used in reconstruction of natural and ASL movements. *l*_1_-norm minimization was used to optimally and sparsely select the synergies. This was similar to the methods in [6]. Briefly, these were the steps involved in the *l*_1_-norm minimization algorithm. Let us assume that, for a subject, *m* synergies were obtained. The duration of the synergies is *t*_*s*_ samples (*t*_*s*_ = 39 in this study). Consider an angular velocity profile of the subject, {**v**(*t*), *t* = 1,…, *T*}, where *T* (*T* = 82 in this study) represents the movement duration (in samples). This profile can be rewritten as a row vector, denoted by **v**_row_ as follows:

(7) $$\begin{array}{c} v_{\text{row}}=\left[v_{1}\left(1\right),\ldots ,v_{1}\left(T\right),\ldots ,v_{10}\left(1\right),\ldots ,v_{10}\left(T\right)\right]. \end{array}$$

Similarly, a synergy **s** ^*j*^(·) can be rewritten as the following row vector:

(8) $$\begin{array}{c} \left[s_{1}^{j}\left(1\right),\ldots ,s_{1}^{j}\left(t_{s}\right),0,\ldots ,0,\ldots ,s_{10}^{j}\left(1\right),\ldots ,s_{10}^{j}\left(t_{s}\right),0,\ldots ,0\right]. \end{array}$$

We add *T* − *t*_*s*_ zeros after each *s*_*i*_^*j*^(*t*_*s*_) (*i* = 1,…, 10) in the above vector in order to make the length of the vector the same as that of **v**_row_. If the synergy is shifted in time by *t*_*jk*_ (0 ≤ *t*_*jk*_ ≤ *T* − *t*_*s*_) samples, and then we obtain the following row vector:

(9) [0,…,0,s1j(1),…,s1j(ts),0,…,0,…, 0,…,0,s10j(1),…,s10j(ts),0,…,0]

with *t*_*jk*_ zeros added before each *s*_*i*_^*j*^(1) and *T* − *t*_*s*_ − *t*_*jk*_ zeros added after each *s*_*i*_^*j*^(*t*_*s*_).

Then, we construct a matrix, *B*, consisting of the row vectors of the synergies and all their possible shifts. The matrix *B* can be viewed as a bank or library of templates with each row corresponding to shifted version of synergy. The first row contains joint angular velocity profiles of each joint of the first synergy. Each subsequent row contains time-shifted versions. Consider

(10) $$\begin{array}{c} B\equiv \left[\begin{array}{ccccccccccccc} s_{1}^{1}\left(1\right) & \cdots & s_{1}^{1}\left(t_{s}\right) & 0 & \cdots & 0 & \cdots & s_{10}^{1}\left(1\right) & \cdots & s_{10}^{1}\left(t_{s}\right) & 0 & \cdots & 0\\ 0 & s_{1}^{1}\left(1\right) & \cdots & s_{1}^{1}\left(t_{s}\right) & \cdots & 0 & \cdots & 0 & s_{10}^{1}\left(1\right) & \cdots & s_{1}^{1}\left(t_{s}\right) & \cdots & 0\\ \vdots & \ddots & \ddots & \ddots & \ddots & \vdots & \cdots & \vdots & \ddots & \ddots & \ddots & \ddots & \vdots \\ 0 & \cdots & 0 & s_{1}^{1}\left(1\right) & \cdots & s_{1}^{1}\left(t_{s}\right) & \cdots & 0 & \cdots & 0 & s_{10}^{1}\left(1\right) & \cdots & s_{10}^{1}\left(t_{s}\right)\\ \vdots & \vdots & \vdots & \vdots & \vdots & \vdots & \vdots & \vdots & \vdots & \vdots & \vdots & \vdots & \vdots \\ s_{1}^{m}\left(1\right) & \cdots & s_{1}^{m}\left(t_{s}\right) & 0 & \cdots & 0 & \cdots & s_{10}^{m}\left(1\right) & \cdots & s_{10}^{m}\left(t_{s}\right) & 0 & \cdots & 0\\ 0 & s_{1}^{m}\left(1\right) & \cdots & s_{1}^{m}\left(t_{s}\right) & \cdots & 0 & \cdots & 0 & s_{10}^{m}\left(1\right) & \cdots & s_{1}^{m}\left(t_{s}\right) & \cdots & 0\\ \vdots & \ddots & \ddots & \ddots & \ddots & \vdots & \cdots & \vdots & \ddots & \ddots & \ddots & \ddots & \vdots \\ 0 & \cdots & 0 & s_{1}^{m}\left(1\right) & \cdots & s_{1}^{m}\left(t_{s}\right) & \cdots & 0 & \cdots & 0 & s_{10}^{m}\left(1\right) & \cdots & s_{10}^{m}\left(t_{s}\right) \end{array}\right]. \end{array}$$

With the above notation, we are trying to achieve a linear combination of synergies that can reconstruct the velocity profiles as in the following equation:

(11) $$\begin{array}{c} v_{\text{row}}=cB, \end{array}$$

where **c** denotes

(12) $$\begin{array}{c} \left[c_{10},c_{11},\ldots ,c_{1K},c_{20},\ldots ,c_{2K},\ldots ,c_{mK}\right], \end{array}$$

where *c*_*jk*_ represents the weight for *j*th synergy, with a shift of *t*_*jk*_. The matrix *B* can be viewed as a bank or library of template functions with each row of *B* as a template. **v**_row_ represents a natural grasp or an ASL postural movement that is being reconstructed. Thus, each time-shifted synergy, represented in *B*, can be used to reconstruct the entire movement.

The following was the optimization problem that was used in selection of synergies in reconstruction of a particular movement:

(13) $$\begin{array}{c} \text{Minimize}\;{||c||}_{1}+\frac{1}{\lambda }{||cB-v_{\text{row}}||}_{2}^{2}, \end{array}$$

where ||·||_1_ represents the *l*_1_ norm, ||·||_2_ represents the *l*_2_ norm or Euclidean norm of a vector, and *λ* is a regulation parameter. Thus, the optimization problem solves for **c**, yielding a sparse selection of synergies that minimizes the difference between the recorded joint angular velocities of a natural grasping movement or ASL postural movement and joint angular velocities of the reconstructed movement.

To evaluate the reconstruction of natural and ASL postural movements, the reconstruction error was determined for each task by

(14) $$\begin{array}{c} \frac{\sum_{i=1}^{n}\sum_{t=1}^{T}{\left[v_{i}^{g}(t)-{\hat{v}}_{i}^{g}(t)\right]}^{2}}{\sum_{i=1}^{n}\sum_{t=1}^{T}v_{i}^{g}{\left(t\right)}^{2}}, \end{array}$$

where ${\hat{v}}_{i}^{g}(t)$ (*t* = 1,…, *T*) is the angular velocity profile of task *g* and finger joint *i* (*i* = 1,…, *n*) reconstructed using a given number of synergies. This yields a ratio between the approximate error and the original angular velocity profile. Thus, an error of 1 represents a squared error of 1 at every time point and every task for each joint. Presented results show normalized error, where an error of zero is minimum error, corresponding to best reconstruction, and an error of 1 is the maximum error corresponding to worst reconstruction.

## 3. Results

**Figure 2:**
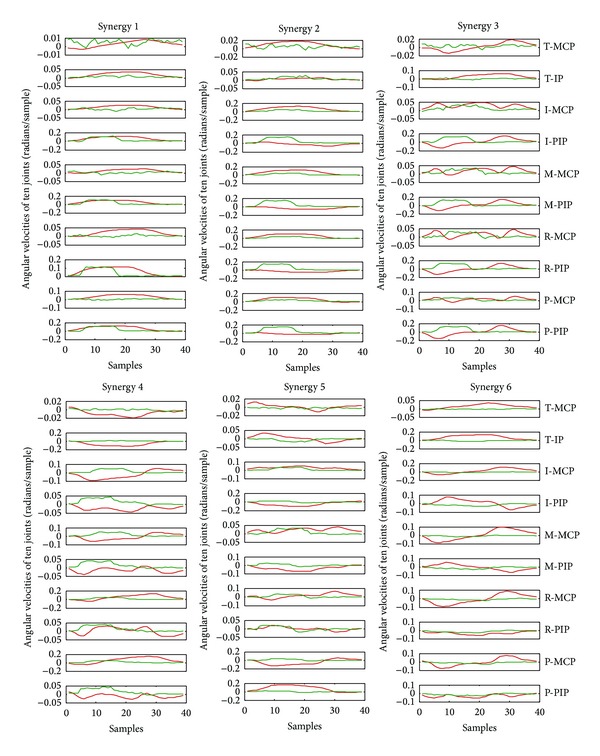
Six kinematic synergies obtained for subject 2 using PCA (red) and ULDA (green). Each synergy is about 0.45 s (39 samples) in duration (data acquired at 86 Hz). T: thumb; I: index finger; M: middle finger; R: ring finger; P: pinky finger; MCP: metacarpophalangeal joint; IP: interphalangeal joint; PIP: proximal IP joint.

**Figure 3:**
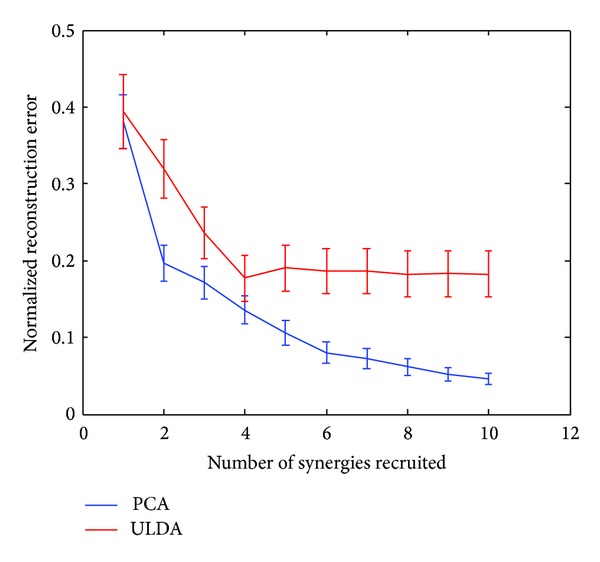
Mean reconstruction errors for PCA (blue) and ULDA (red) using up to 10 synergies are shown. Reconstruction errors for natural grasping movements were less for PCA derived synergies than ULDA derived synergies. Reconstruction errors for ULDA plateaued after 5 synergies.

**Figure 4:**
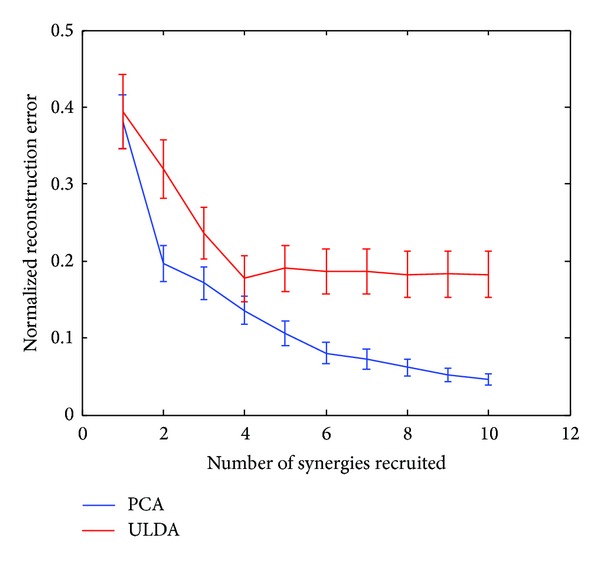
Reconstruction errors for ASL postural movements were similar for both PCA and ULDA up to 5 synergies, after which ULDA errors plateaued and PCA errors decreased.

PCA and ULDA described in Section 2.3 were used to derive kinematic synergies from preprocessed hand joint angular velocities during rapid grasps. The top six significant synergies derived from PCA and ULDA were used in reconstruction of natural movements and ASL postural movements.  Figure 2 shows a representative example of six synergies derived from PCA and ULDA for one subject. Synergies 1 and 2 derived from PCA show broad, bell-shaped velocity patterns while synergies 1–5, derived from ULDA, especially in PIP joints, show distinctly different patterns. Interestingly, synergies derived from ULDA show an initial movement delay which is consistent in PIP joints for synergies 1–5. In both methods, velocity patterns for higher-order synergies are characterized by multiple submovements. Extension is first seen in synergy 2 derived from PCA and synergy 6 derived from ULDA. In terms of joint angular velocity patterns, kinematic synergies further reveal relationships found between joints. PIP joints reach a greater velocity than MCP joints in synergies 1 and 3. Conversely, MCP joints reach a greater velocity than PIP joints in synergies 2 and 4. The same inverse relationship, where PIP joint reach a greater velocity than MCP joints, is found in synergy 1 derived from ULDA. However, only synergy 5 shows a movement where MCP joints have greater velocities than PIP joints. These results encourage further analysis on the importance of each synergy. In order to determine how the number of synergies used in reconstruction affects performance of each method, we calculated reconstruction errors for up to 10 synergies (Figures 3 and 4). In both of these figures, we can see the performance of ULDA plateaus after 4 synergies. Reconstruction error continues to decrease for PCA as more synergies are used. It is evident that PCA performs better than ULDA in natural grasp reconstruction. However, reconstruction errors of ASL postural movements are similar for both methods until 5 synergies are used, after which the performance of ULDA plateaus and PCA error decreases (Figure 4).

**Figure 5:**
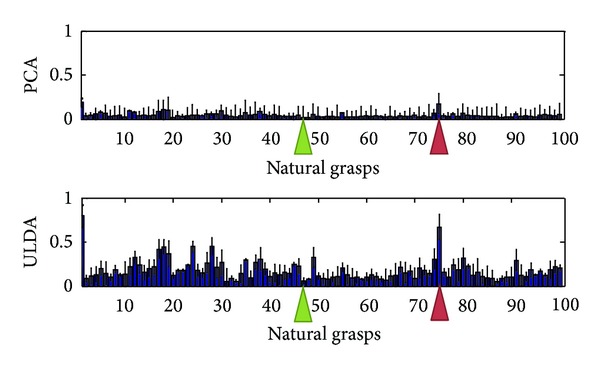
The mean reconstruction errors for 100 natural grasping movements using PCA and ULDA across all subjects. PCA performed better than ULDA. Reconstruction error is computed by (14). Two tasks corresponding to best and worst reconstruction errors were indicated by green and red arrows, respectively. These were further illustrated in Figure 7.

**Figure 6:**
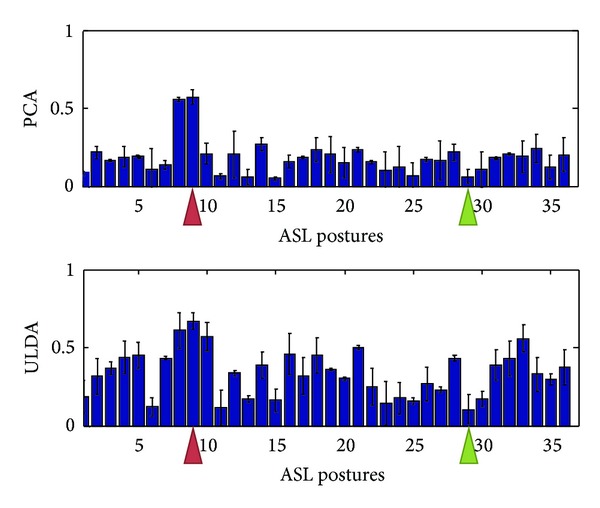
The mean reconstruction errors for 36 ASL postural movements using PCA and ULDA across all subjects. PCA performed better than ULDA. Reconstruction error is computed by (14). Two tasks corresponding to best and worst reconstruction errors were indicated by green and red arrows, respectively. These were further illustrated in Figure 8.

**Figure 7:**
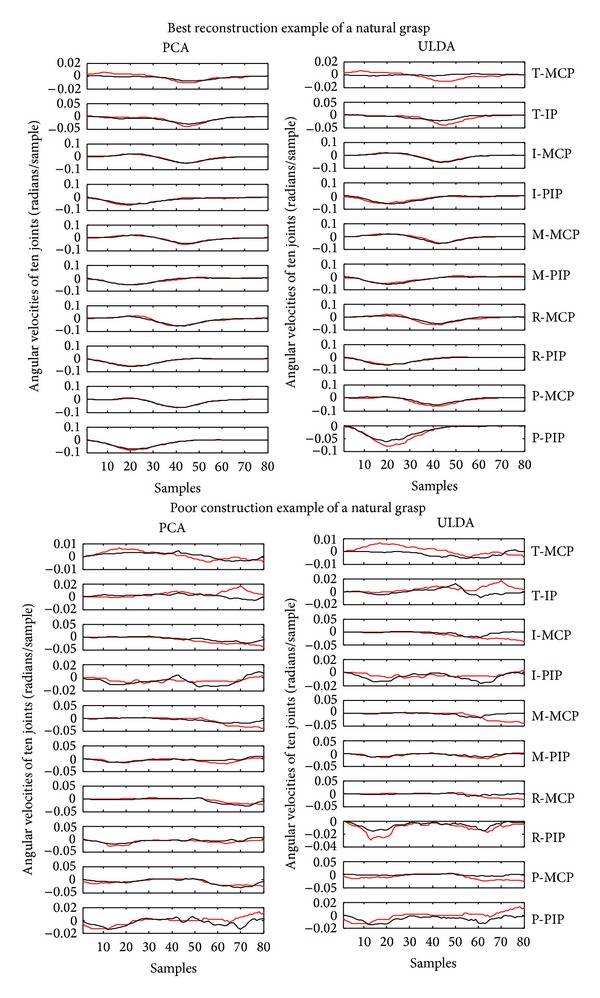
Top: the lowest reconstruction error for both methods in natural grasping movement reconstruction was found in grasping task number 47. Its reconstruction is shown using PCA and ULDA. Bottom: the highest reconstruction error for both methods was found in grasping task number 75. Its reconstruction is shown using PCA and ULDA. T: thumb; I: index finger; M: middle finger; R: ring finger; P: pinky finger; MCP: metacarpophalangeal joint; IP: interphalangeal joint; PIP: proximal IP joint.

**Figure 8:**
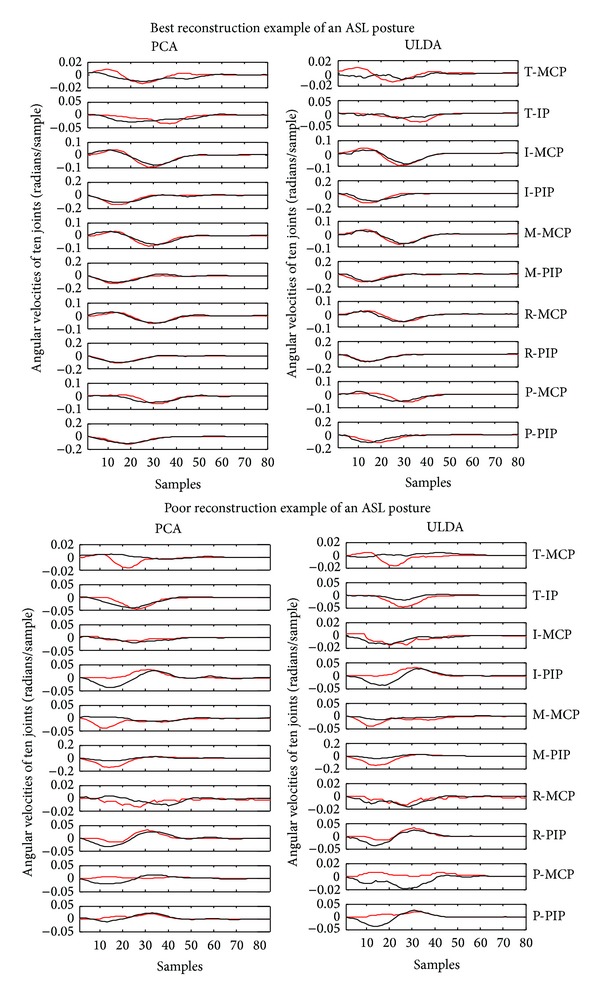
Top: the lowest reconstruction error for both methods in ASL postural movement reconstruction was found in ASL postural task number 29. Its reconstruction is shown using PCA and ULDA. Bottom: the highest reconstruction error for both methods was found in ASL task number 9. Its reconstruction is shown using PCA and ULDA. T:thumb; I: index finger; M: middle finger; R: ring finger; P; pinky finger; MCP: metacarpophalangeal joint; IP; interphalangeal joint; PIP: proximal IP joint.

Using the optimization algorithm, all natural grasps and ASL postural movements were reconstructed.  Figure 5 shows the mean reconstruction errors for 100 natural movements across 10 subjects. Error bars indicate the standard deviation across 10 subjects. Similarly, Figure 6 shows the mean reconstruction errors for 36 ASL postural movements. Error bars indicate the standard deviations across 10 subjects. PCA had the best overall performance with mean reconstruction errors of 0.08 and 0.2002, for natural grasping and ASL postural movements, respectively. ULDA has reconstruction error of 0.1867 and 0.3147 for natural grasping and ASL postural movements, respectively. Green and red arrows indicate movements that had the best and worst reconstructions for both methods, respectively. These reconstructions are shown in Figures 7 and 8.  Figure 7 (top) shows a reconstruction of grasping task number 47, which had the lowest reconstruction errors for both methods; we observe that both methods were able to reconstruct a natural grasp with minimal error; however, ULDA was unable to replicate joint extension in the thumb and pinky PIP joints.  Figure 7 (bottom) shows a reconstruction of a natural grasping task number 75, which had the highest reconstruction error for both methods. In this task, the recorded movement (red) lacks smooth velocity patterns and is also characterized by lower velocities. This type of movement was also seen in natural grasping tasks numbered 1, 12, 17, 18, 38, 49, and 80. Both methods were unable to replicate this type of movement, although PCA had lower errors. Reconstruction errors of ASL postural movements were greater than those of natural grasps for both methods.  Figure 7 (top) shows the best reconstruction of an ASL postural movement (task number 29). PCA was unable to replicate the magnitude of velocity while ULDA was unable replicate the temporal structure of each joint angular velocity profile. ASL postural movement task number 9 had the highest reconstruction error and is shown in Figure 8 (bottom). Both methods were unable to replicate extension that was not consistent across joint types.

## 4. Discussion

Anatomical, neural, and functional coupling in the human hand suggests that all possible hand postures from everyday activities lie in a lower-dimensional subspace that can be characterized by kinematic synergies [5]. The synergies in this paper were derived from grasping kinematic data and were tested for generalizability on ASL postures. Principal component analysis was performed on the posture matrix or in other words on the high-dimensional space of 100 postures, meaning the variables here are postures and the observations are joint angular velocities for these postures. The synergies are principal components that are expressed as weighted linear combinations of these 100 postures. The weights are derived from eigen vectors. As principal components represent the axes aligned with the directions of maximum variance, the synergies are the most commonly found joint angular velocity patterns used during the 100 postural tasks. Six synergies were found to account for 95% of the variance. Linear discriminants on the other hand, as candidates for synergies, represent postures that stand distinct among groups in 100 postures. The groups were formed based on the nearest neighbors computed by measuring the Euclidian distances between postures.

Results show that PCA outperforms ULDA in reconstruction of natural grasping movements and ASL postural movements. Does that mean that PCs are optimal low-dimensional representations of hand movements and hence ideal candidates for synergies? Linear discriminants, although representing the six most distinct joint angular movement velocity patterns, may not be ideal candidates for synergies as they are not capturing the commonly found joint angular velocity patterns. Interestingly, previous studies exploring muscle synergies have found distinct patterns through clustering to be more effective than PCA in synergy extraction. Krouchev et al. [19] found that distinct muscle groups characterize each synergy extracted from cat locomotion movements via associative clustering. Ajiboye and Weir [13], using NMF, also found that population synergies extracted from hand movements (ASL postures) were dominated by a single muscle while subject-specific synergies involved coactivation of multiple muscles. Although, in muscle space, distinct groups of muscles may better characterize movement, this phenomenon does not carry to kinematic space. PIP joint angular velocities showed a distinct waveform that could be seen in synergies 1–5 extracted from ULDA, while the 6th synergy shows a similar joint angular velocity profile across all PIP and MCP joints. ULDA failed to capture the relationship between all MCP and PIP joints in multiple synergies, as indicated in the synergy patterns (Figure 2). Natural coupling between fingers, as a result of single or multiple muscle activations, is captured by both PCA in synergies 1–4 and ULDA in synergies 1–6 because all 4 fingers MCP show similar velocity patterns. Synergies 5 and 6, derived from PCA, however, do not represent physiological couplings but may characterize less commonly used and more independent movements during grasping. For example, synergy 5 derived from PCA shows R-PIP extension during M-PIP flexion, which does not seem intuitive but may be a result of more complex grasps.

Although PCA derived synergies outperform ULDA derived synergies in ASL postural movement reconstruction, there is still an increase in error for both during reconstruction of ASL postural movements. One possible source for increased error in ASL reconstruction is that ASL postures require extreme decoupling of fingers. For example, the three distal fingers are anatomically coupled and grasping movements do not usually force them apart. However, in ASL, number 8 (task number 9) requires the subject to flex the middle finger with the assist of the thumb, while consciously trying to extend the ring finger. Since the middle and ring fingers are naturally coupled, synergies are unable to capture such an extreme movement, resulting in highest reconstruction error (Figure 6).

In summary, two contrasting dimensionality reduction methods PCA, that maximizes variance and LDA, that discriminates variance, have been used to extract synergies. The contribution of linear discriminants plateaued beyond 5 synergies, but the contribution of principal components continued beyond six synergies. PCA outperformed LDA in reconstruction of natural grasping and ASL postural movements. The shape, smoothness, and representations of natural joint couplings found in joint angular velocity profiles may have contributed to differences in reconstruction errors. Certain types of natural grasps, namely, those characterized by irregular and noisy velocity profiles, yielded higher reconstruction errors for both methods. ASL postural movements included unnatural flexion and extension of fingers that are not involved in the activities of daily living. For this reason, both PCA and LDA had greater errors in reconstruction of ASL movements. In the future, combinations of linear and nonlinear dimensionality reduction methods will be used in reducing the dimensionality of hand movement kinematics.
